# Using Microbiome-Based Approaches to Deprogram Chronic Disorders and Extend the Healthspan following Adverse Childhood Experiences

**DOI:** 10.3390/microorganisms10020229

**Published:** 2022-01-21

**Authors:** Rodney R. Dietert, Janice M. Dietert

**Affiliations:** 1 Department of Microbiology and Immunology, Cornell University, Ithaca, NY 14853, USA; 2 Performance Plus Consulting, Hereford, AZ 85615, USA; maninmirror90@gmail.com

**Keywords:** microbiome, adverse childhood experiences (ACEs), gerobiotics, microimmunosome, healthspan, circadian rhythms, sleep disorders, noncommunicable diseases and conditions (NCDs), chronic disorders, early life programming

## Abstract

Adverse childhood experiences (ACEs), which can include child trafficking, are known to program children for disrupted biological cycles, premature aging, microbiome dysbiosis, immune-inflammatory misregulation, and chronic disease multimorbidity. To date, the microbiome has not been a major focus of deprogramming efforts despite its emerging role in every aspect of ACE-related dysbiosis and dysfunction. This article examines: (1) the utility of incorporating microorganism-based, anti-aging approaches to combat ACE-programmed chronic diseases (also known as noncommunicable diseases and conditions, NCDs) and (2) microbiome regulation of core systems biology cycles that affect NCD comorbid risk. In this review, microbiota influence over three key cyclic rhythms (circadian cycles, the sleep cycle, and the lifespan/longevity cycle) as well as tissue inflammation and oxidative stress are discussed as an opportunity to deprogram ACE-driven chronic disorders. Microbiota, particularly those in the gut, have been shown to affect host–microbe interactions regulating the circadian clock, sleep quality, as well as immune function/senescence, and regulation of tissue inflammation. The microimmunosome is one of several systems biology targets of gut microbiota regulation. Furthermore, correcting misregulated inflammation and increased oxidative stress is key to protecting telomere length and lifespan/longevity and extending what has become known as the healthspan. This review article concludes that to reverse the tragedy of ACE-programmed NCDs and premature aging, managing the human holobiont microbiome should become a routine part of healthcare and preventative medicine across the life course.

## 1. Introduction

Early life adverse environmental, emotional, and physical experiences can have a heightened impact on the development of tissues, organs, and physiological (systems biology) units when compared with similar exposures in the adult. This was codified in the mantra that children are not simply small adults and never should be treated as such. When benchmark maturational events are disrupted via chemicals, drugs, food, food additives, physical or emotional exposure, inappropriate maturation and subsequent dysfunction of the body’s systems are likely. In effect, human systems are readily programmed for later life dysfunction and chronic disease when the fetus, newborn, infant, and adolescent are inadequately protected during critical windows of developmental vulnerability. This special vulnerability in early life and the need for special protections of the young have been described in a series of papers and reports [1,2,3,4,5] that contributed to what became known as the scientific field of Developmental Origins of Health and Disease (DOHaD).

Two developments within the DOHaD umbrella during the past decade are changing how we view the ongoing epidemic of noncommunicable diseases and conditions (NCDs). First, the microbiome is now recognized as central to the maturation of human systems biology units (e.g., gut–brain, microimmunosome, gut–hypothalamic–pituitary–adrenal (HPA) axis, gut–liver, gut-bile acid metabolism). Disruption of microbiome maturation in early life invariably results in disrupted systems biology units and elevated risk of childhood and adult NCDs. Unless the dysbiotic microbiome is addressed in dealing with systems biology malfunction and NCDs, the epidemic of NCDs is likely to continue unabated.

Second, physical and emotional trauma in early life (often called adverse childhood experiences, ACEs) damage not only organ/tissue development (e.g., brain, gut, immune) but also microbiome status, thereby, impacting the risk of a diverse range of later-life, comorbid NCDs. In many ways, physical and emotional “toxicity” for the microbiome and multiple systems biology units have been understudied and potentially underappreciated. While adverse effects of ACEs on the brain are important, those changes do not occur in a vacuum nor are they the only mis-programming that occurs in early life.

This review article is not intended to provide an exhaustive review on any single disciplinary-related topic (e.g., the HPA axis). Instead, it draws primarily upon the last five years of research to examine the intersection of the microbiome and systems biology units as it pertains to ACE-induced programming. Specifically, the article examines: (1) the prevalence of ACEs, (2) the inadequate protection of the young from physical and emotional abuse (including child trafficking), (3) the significance of premature aging and NCDs resulting from ACEs, (4) the interconnections between microbiome status, circadian rhythms, sleep quality, NCDs including depression, and longevity, and (5) the opportunities to broaden historic organ-centric approaches to focus on microbiome-systems biology correcting solutions. The importance of microbiome status in early life cannot be overemphasized. This was captured in a recent paper describing the connection of infant antibiotic exposure to risk of a broad range of childhood NCDs [6].

## 2. Adverse Childhood Experiences and the Microimmunosome

Adverse childhood experiences (ACEs) in early life are among the most devasting physiological and microbiological programming events that exist. During critical windows of vulnerability, these adverse experiences can significantly increase the disease and disability burdens across the lifespan. One of the early studies that utilized the term adverse childhood experience was a U.S. Centers for Disease Control and Prevention (CDC)-Kaiser Permanente study [7].

There is considerable debate about the full range of meaningful events that constitutes ACEs, and this has resulted in a general lack of standardization for studying the effects of adverse childhood experiences on future health [8]. One recent example was utilized by Lin et al. [9] in their study in China and includes the following 12 ACEs: physical abuse, emotional neglect, household substance abuse, household mental illness, domestic violence, incarcerated household member, parental separation or divorce, unsafe neighborhood, bullying, parental death, sibling death, and parental disability.

Regardless of the difference in what spectrum of ACEs were included in various studies, it was clear that these ACEs, including events such as childhood trauma, readily program the child for NCDs and additional conditions such addictive behaviors [10,11]. Examples of ACE DOHaD-like programming of later-life NCDs include: asthma [12], obesity and diabetes [13], cardiovascular disease [14], neurobehavioral disorders [15], and cancer [16]. Furthermore, beyond the scope of this present review, ACEs can transmit elevated intergenerational risk for chronic disorders across generations [17,18]. The CDC developed an ACE pyramid to visually reflect the diverse outcomes of ACEs across the lifespan [19]. However, as with much of public health [20], it does not capture either the outcomes or interrelationships pertaining to the microbiome.

Based on data from the most recent survey involving 25 states, the U.S. CDC estimated that approximately 61% of adults had at least one adverse childhood experience with approximately 16% reporting four or more experiences [21]. Unsurprisingly, the cost in human capital and even national economic sustainability is staggering. A recent survey of European countries estimated that the economic burden of ACEs amounted to more than 120 billion US dollars per annum for a country such as Germany and ranged up to 6% of Ukraine’s gross domestic product [22].

While an increased focus needs to be brought to bear on preventing these experiences, we have only begun to address the role of microorganisms particularly in conjunction with the mucosal immune system in both the disease state and in therapeutic applications. The present review is not intended to be a comprehensive tome covering all aspects of ACE-induced diseases. Instead, it focuses on six microimmunosome-dependent adverse outcomes: noncommunicable diseases and their comorbidities, circadian rhythm disruption, early-onset aging/shorter lifespan, predispositions to addiction, mood disorders, and sleep disorders.

ACE-induced chronic disorders have often been medically treated at the level of the dysfunctional tissues or organs. Examples would be a focus on (1) the hypothalamic–pituitary–adrenal (HPA) axis for cortisol regulation, (2) the brain for mood disorders, (3) or the target tissue in which a given NCD arises (e.g., the lungs in pediatric and adult asthma). But these limited, end-result approaches can fail to restore crucial master regulations involving core cyclic rhythms, broad systems biology units, and the multiple levels of microbiome control over human systems biology (e.g., the microimmunosome, the gut–immune–brain axis). The following sections are designed to provide a broader view of potential ACE-driven programming and deprogramming beginning with the master controller of premature aging of both human systems biology and the microbiome.

## 3. The Range of ACE-Programmed Chronic Diseases and Disorders

ACE-induced programming has a broad range of adverse outcomes that appear across childhood and in the aging adult. Table 1 [7,9,23,24,25,26,27,28,29,30,31,32,33,34,35,36,37,38,39,40,41,42,43,44,45,46,47,48,49,50,51,52,53,54,55,56,57,58,59] illustrates the extensive impact that these experiences in isolation or combination can have across multiple different systems biology units. It is emphasized that while these diseases and disorders emerge in different organs and physiological systems, their dysfunctional origins are either controlled by or significantly influenced by the genes, metabolic activity, signaling, and epigenetics regulation from the human microbiome. With this in mind, effective approaches to correcting and rebalancing the early life damages to the human holobiont need to include not only the microbiome but also the larger inflammation-controlling unit, the microimmunosome.

While many approaches to treating ACE-induced conditions have focused on the downstream set of imbalances and symptoms (e.g., HPA axis, brain/neurological/psychological treatment), the inclusion of a more upstream and comprehensive approach that includes Microbiome First medicine is needed.

## 4. ACE-Programmed Misregulated Inflammation and Specific NCDs

Among the devastating disease-promoting programming that results from ACEs is the development of chronic underlying inflammation and a spectrum of specific comorbid NCDs. As discussed in prior reviews [60,61], the accumulation of NCDs, polypharmacy and in many cases caregiver needs is a path that increases medical needs and significantly reduces quality of life. One problem with these ACE-linked outcomes is that to date, both medicine and public health have produced few cures for NCDs, and the epidemic of these conditions continued across decades making NCDs the leading cause of death (estimated at 71% of all deaths) globally [62]. The lack of cures and emphasis to date on symptom management has permitted the inflammation-connected expansion of comorbidity and polypharmacy associated with aging [61]. The same pattern has arisen with ACE-connected NCDs and other imbalances during the aging process. However, the symptom driven healthcare approaches to NCDs to date have largely excluded microorganisms and the microbiome from priority consideration [61].

Table 2 [12,16,63,64,65,66,67,68,69,70,71,72,73,74,75,76,77,78,79,80,81,82,83,84,85,86,87,88,89,90,91,92,93,94] illustrates examples of the specific NCDs to date that are connected to ACE through elevated prevalence. Of note is the fact that they span allergic, autoimmune, metabolic, and inflammatory diseases including diabetes and heart disease as well as neurodevelopmental and neurodegenerative diseases.

As discussed by Dietert [61], childhood onset NCDs such as asthma and metabolic syndrome (e.g., obesity, diabetes) are entryway conditions that are connected to a large number of later-life comorbidities via the inflammation-NCD cascade. ACE-programmed NCDs and physiological changes present added challenges since behavioral programming drives this cohort toward exposures that further increase the risk of comorbid diseases. Among these are a predisposition for substance misuse/abuse and risky behaviors that could promote additional microbiome dysbiosis, degraded colonization resistance, and significant advantages for pathobionts to produce infections.

## 5. Additional Outcomes of ACEs with Microbiota Regulation

Two adverse outcomes received prior recent reviews within the Microbiome First series of papers. These concerned the capacity of the microbiome to regulate pain [95] and both the occurrence of substance abuse and the likelihood of successful withdrawal [20].

### 5.1. Pain

One of the characteristics of ACEs and programmed systems biology alteration is the increased likelihood of later-life chronic pain. Several studies have specifically examined the relationship between childhood adverse experiences and the appearance and persistence of adolescent and adult pain. Examples of recent ACE-pain studies are shown in Table 3 [40,41,42,43,44,96].

As recently reviewed in Dietert and Dietert [95], microbiome adjustment including treatment with probiotics and prebiotics has the capacity to alter a variety of different types of pain. These alterations include changes in the perception of pain and in its persistence at various life stages. A sample of the research papers and review analyses are included here [97,98,99,100,101,102,103,104,105,106]. While the focus of this present review is on ACE-programmed dysfunction in holobiont regulatory cycles that lock in both NCDs and systems biology dysfunction, the reach of microbiome controls includes quite specific endpoints that impact quality of life functional capacities. This is one of the reasons that management of the early life both before and after ACE is the key to improved resiliency. In the later section discussion of circadian rhythms, it will become clear that disrupted circadian clocks are tightly associated with increases in pain. Therefore, ACE-associated damage to the more global body cycles appears to be one way for pain perceptions and threshold to change for the worse.

### 5.2. Substance Misuse/Abuse

ACEs have a strong association with substance abuse and addiction. Multiple systems are likely involved and changes in the reward system are thought to play a major role [107,108,109]. As with other programmed changes, substance abuse carries its own additional risks as aging progresses (e.g., the damages created by prolonged tobacco, alcohol, and drugs abuse).

Table 4 [37,46,110,111,112,113,114] illustrates examples of substance abuse connected to ACE. Given the knowledge that this is a significant risk, preventative measures that include the microbiome and protection against dysbiosis not only reduce the risk of substance abuse, but also canaid in protection against impediments to withdrawal. This was previously discussed in two recent reviews [20,95].

## 6. At the Epicenter of NCDs

In an early, collaborative article published in Environmental Health Perspective examining the comorbidities arising from immune dysfunction-inflammation-promoted NCDs [115], we noted a specific pattern of NCDs and related dysfunctions that were shared by most childhood-young adult onset NCDs. These were: sleep disorders, depression, sensory disorders, and cardiovascular disease. Of course, the question then was why were these specific conditions shared as comorbidities by most of the NCDs examined in our publication? Now more than a decade later, the answer would seem to be at hand. Sleep disorders, depression, and atherosclerosis are intimately linked to microbiome status, misregulated inflammation, and disruptions of circadian rhythms.

## 7. Circadian Rhythms

Circadian rhythms are recognized as not simply a novel occurrence within the human body but, more significantly, a key cycle that: (1) spans the operation of systems biology units [116,117], (2) connects the microbiome with those units [118], and (3) ultimately can result in healthy metabolism and physiology [119] or disease [120,121,122].

Table 5 [123,124,125,126,127,128,129,130,131,132,133,134,135,136] illustrates examples of NCDs and other disorders that are intimately connected to a disruption in circadian rhythms. Because of the tight interconnects that exist between the circadian clock, the microbiome, and systems biology homeostasis or dysbiosis, it is sometimes difficult to distinguish what elements are actually the penultimate controllers. When it comes to managing health and disease, the interconnections are probably what matters most. For example, aligning circadian rhythms may not inherently repair microbiome dysbiosis and/or a compromised gut barrier. On the other hand, microbiome rebiosis and adjustments to the microimmunosome, gut–immune–brain axis, or HPA axis may only endure if not repeatedly undermined by a circadian rhythm defect. As will become apparent in the following sections, attention to both factors in combination is likely to be a more successful health promoting strategy.

In support of the idea of a combined effort to promote a healthy microbiome, downstream systems biology units (e.g., the microimmunosome) and balanced circadian rhythms, Table 6 illustrates recent studies demonstrating the interconnectivity between microbiome status and circadian rhythms. Importantly, as Microbiome First applications become increasingly utilized across medicine and public health [20,61], the status of the circadian clock will be a critical co-factor in successful outcomes.

## 8. Sleep and Microbiota

Insomnia and other sleep disorders are a prevalent outcome not only of adverse childhood experiences but also of many NCDs [115]. Sleep disorders are very interconnected to circadian disruption, specific NCDs, pain and inflammation, and microbiome dysbiosis. The tight interconnectivity between these can make the cause–effect relationship challenging to determine. A recent review by Kang et al. [143] examined the gut microbiome as a target for adjunct therapies to address insomnia.

Defining the tipping point causes in sleep disruption constitutes a large part of ongoing research. However, it is already clear that microbiome dysbiosis can lock in NCDs, systems biology dysfunctions (e.g., neuro-brain, HPA axis, bile acid metabolism, microimmunosome), and sleep disorders. Correcting imbalances within the microbiome is significant if healthy sleep-circadian rhythm patterns are to be restored and maintained. We can no longer afford to ignore the microbiome in NCD-systems biology oriented therapies [61]. Table 7 [144,145,146,147,148,149,150,151,152,153,154,155] illustrates the impact of microbiome status on sleep.

## 9. Inflammation, Oxidative Stress, and the Longevity Cycle

As shown in Table 1 one of the outcomes of adverse childhood experiences is premature aging including shortened telomeres. At the heart of both ACEs and NCDs is misregulated inflammation. Chronic unresolving tissue inflammation even at low levels creates increased oxidative damage to a variety of macromolecules, tissue pathology, and eventually NCDs ranging from asthma, cardiovascular, metabolic, inflammatory bowel, and psoriasis diseases to cancer. It has been suggested that aging is a multifactorial, multisystem event that is best characterized by a network of biomarkers ranging from functional to molecular in nature. In fact, Wagner et al. [156] listed 22 different biomarkers that capture the different level of potential evaluation of aging.

One of the biomarkers reflecting the persistent inflammation, increased oxidative stress and premature aging is the shortening of chromosomal telomeres [157]. Telomere length is thought to reflect the number of cell cycles that a population of cells can undergo. When certain cells can no longer divide, the body is incapable of maintaining critical functions.

Table 8 [158,159,160,161,162,163] illustrates examples of the relationship between inflammation, oxidative damage and telomere length. It should be noted that efforts to minimize the symptoms of NCDs rather than correcting underlying systems biology defects (e.g., unresolving tissue inflammation) have two very negative outcomes. First, comorbid NCDs and increasing polypharmacy are not abated by the symptom management based on the pattern of the last several decades [61]. Second, the shortened telomeres and increased oxidative stress from the unrelenting misregulated inflammation means that the patient will invariably have a shortened life compared with a cohort that is disease free (i.e., cured). Symptom-only management should be viewed at best as a transient goal when compared to disease-free, life-extending therapeutic outcomes.

## 10. The Immunological Epigenetic Clock of the Microimmunosome

One of the recent findings that pertains to longevity concerns the relationship between immune senescence and aging. As we age quite specific changes occur in the immune system that affect not only the risk of NCDs but also the relationship between the tissue-distributed immune system and organ homeostasis. As the immune system fails, ultimately it impacts our organs and physiological systems [164].

A recent discovery is that the immune system has its own epigenetic clock and by youthanizing the immune system, the effect spreads across our other systems biology units. Fahy et al. [165] published results of reversing the path toward immune senescence by tapping regenerative processes within the thymus and bone marrow. Because the overriding theme of ACEs is premature aging with chronic inflammation and oxidative stress, the capacity to initiate multi-system age regression within the microimmunosome and to potentially correct misregulated inflammation is very promising.

## 11. Gerobiotics as a Microbiota-Based Anti-Aging/Healthspan Strategy

Systems biology distributed changes that occur with adverse childhood experiences can also occur with early life chemical and drug toxicity or inadequate seeding and feeding of the infant microbiome. These interconnected changes are centered around a pattern of chronic inflammation, immune-, mitochondrial-, telomere-, and microbiome aging and the NCD comorbid cascade. The weakness in providing better protection of a major cohort of early life programmed children is to selectively work on only one aspect or biomarker of the complex premature aging pattern. The overall goal in looking toward gerobiotics is not simply increased longevity but rather extending the healthspan, the number of healthy years within a person’s life [166].

The present review emphasizes the important role of using microbiota, their metabolism, signaling and epigenetic control of multiple physiological systems to facilitate an unwinding of the DOHaD installed NCD-rich, premature death programming. Table 9 [167,168,169,170,171,172,173,174,175,176,177,178,179,180,181,182,183,184,185,186,187,188,189,190,191,192,193,194] provides some of these examples.

## 12. Discussion

This review is novel in its consideration of early life programming resulting from Adverse Childhood Experiences. Not only are the specific, extensive ACE-programmed childhood and adult onset NCDs (i.e., chronic diseases and conditions) presented but also the connections between ACE and the destruction of microbiome balance, the circadian rhythm cycle, and the healthy portion of the longevity cycle (i.e., the healthspan). The presented material within the tables and narrative illustrates a key point: The microbiome and the circadian rhythm cycle are master regulators of the very diseases that are the number one cause of global death [W62]. Longevity and the healthspan are outcomes of the master regulation. This is significant because both the prevention of ACE-programmed NCDs and the therapeutic plans that follow NCD diagnoses often fail to include the correction of microbiome and circadian cycle/sleep defects. Yet, disease “cures” become less likely when the master regulators are left in a dysfunctional state.

Dietert discussed this very issue relative to the microbiome dysbiosis in two recent review articles [3,61]. Additionally, the significance of including the circadian cycle [119,120,129] and the longevity cycle [195] in healthcare/public health plans has been stressed in a number of recent publications. Much as the microbiome has bidirectional communication with the immune system through the microimmunosome [60,61], microbiota and circadian cycles are involved in cross-talk [196]. Therefore, both need to be considered together much like one would approach a systems biology unit.

For the longevity cycle, prior treatments have largely included dietary recommendations [197,198]. However, a larger collection of factors is now being considered. For example, numerous factors have been examined for anti-aging activity including 17β-Estradiol, melatonin, metformin, rapamycin, coenzyme Q10, N-acetyl cysteine, and vitamin C based on protection against stem cell senescence [199]. At least one existing drug, metformin, a plant derived drug, has shown considerable promise for its capacity to serve as a longevity drug [200,201]. Numerous down-stream effects have been seen with metformin treatment, making it clear that many benefits from this drug arise via its direct effects on the microbiome [202,203,204].

As many investigators have pointed out when examining anti-aging/longevity initiatives, the ultimate goal is not simply more years added to a disease-filled life but actually more years spent in a healthy life. This is how the concept of the “healthspan” has emerged [205,206]. It is healthy longevity that is the sustainable healthcare prize and not a few extra polypharmacy-riddled, low quality of life days added to our current multimorbid final years. What is clear is that reduced prevalence of NCDs, balanced circadian rhythms, effective pain management, effective sleep quality, and a longer healthspan can only occur if supported by a healthy microbiome across the life stages [3,61,207,208,209,210].

**Figure 1:**
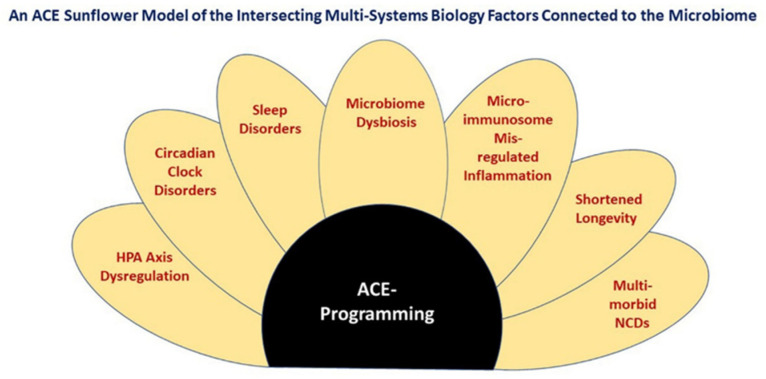
Shows a Venn Diagram in the form of sunflower ray florets connected to Adverse Childhood Experience (ACE)-programming depicted as the central disc floret. The multi-system dysfunctions and dysbioses arising from ACEs are interconnected with each other and greatly affected by the status of the microbiome. For this reason, a lifelong solution to the chronic disorders arising from ACEs requires a holobiont-wide strategy.

The microbiome can regulate overlapping systems biology units, and this combined status can affect the risk of NCDs. To capture the relationships between the microbiome, the systems biology units and ACE programming, Figure 1 illustrates a “sun-flower” model of this interconnectivity.

Inattention to microbiome status and incomplete therapeutic efforts can undermine necessary corrections when it comes to sleep regulation, circadian rhythms, the microimmunsome, anti-aging factors, and the HPA axis. For this reason, the more holobiont-focused the approach, the better.

Finally, one of the significant contributors to ACEs and the resulting adverse health challenges that follow is child trafficking [211]. This form of modern slavery has been difficult to accurately estimate at a global or even a national level [212]. There is a bi-directional relationship between child trafficking and ACEs. In a study in Florida, Reid et al. [213] found that prior childhood adversities increased the likelihood that a given child would be trafficked. But pediatric associations have recognized that child trafficking itself presents a critical health threat [214,215,216]. It is clear that preventing ACEs and child trafficking is the highest priority. Revelations concerning child trafficking activities may help to raise public awareness and reduce its prevalence [217,218]. When prevention fails, microbiome-driven, multi-systems biology approaches offer a comprehensive strategy to reduce the burden of ACE-programmed NCDs.

## 13. Conclusions

Adverse childhood experiences (ACEs) including the trafficking of children represent a significant health risk. ACEs program microbiome dysbiosis, increased risk of specific NCDs (e.g., depression), increased chronic inflammation, increased oxidative stress, increased mitochondrial dysfunction, disrupted circadian rhythms, shortened telomeres, reduced longevity, and a greatly abbreviated healthspan. A first priority should be to keep children out of harm’s way as much as possible and to better protect children from those experiences that are preventable. They should never face preventable trauma. However, it is also important to enhance resiliency and better prevent and treat chronic disorders, including NCDs.

A healthy microbiome is a route to provide enhanced resiliency in childhood and to deprogram both comorbid NCDs and multiple systems biology dysfunctions. The circadian rhythm cycle and sleep quality respond to and affect many biological functions, longevity, and microbiome status. But it is clear that healthy circadian rhythm, sleep, as well as effective longevity cannot persist in the face of: (1) microbiome dysbiosis, (2) damage to the microimmunosome, and (3) the all-too-common outcome of misregulated inflammation.

Ensuring both an optimized microbiome and balanced microimmunosome should be top priorities for ACE deprogramming of the human holobiont. Otherwise, efforts to correct ACE-based problems using classical disciplinary-based approaches are likely to produce underwhelming outcomes when viewed across the lifespan. This present review illustrates the benefits of utilizing a microbiome-driven, systems biology approach to unwind the devastating lifelong programming established through multiple adverse childhood experiences.

## Figures and Tables

**Table 1:** Examples of Childhood and Adult Outcomes Linked with Adverse Childhood Experiences.

Conditions/Attribute	Details	References
Compromised immune system	Chronic inflammation	[23]
Compromised immune system	Elevated inflammation in women: Late menopause timed.	[24]
Compromised immune system	Early senescence in natural killer cells.	[25]
Elevated risk of NCDs	Elevated risk for the vast majority of the NCDs connected to ACEs.	[7,9,26,27]
Premature Aging/Shorter Lifespan	Epigenetics changes; Inflammaging; Shorter telomere length.	[28,29]
Sleep disorders	ACE-connected sleep disorders reported both in adolescents and adult; various sleep disturbances; examined via longitudinal study and other methods.	[30,31,32,33]
Circadian rhythm disruption	Often measured as disrupted circadian cortisol secretion or secondarily as clock gene expression.	[34,35,36]
Substance Misuse/Abuse/Addiction	Smoking/Alcohol/Drugs	[37,38]
Additional Risky Behavior	Early sex, multiple partners/early pregnancy	[39]
Chronic pain	Both adolescent and adult onset; cortisol levels can be a biomarker.	[40,41,42,43,44]
Unfavorable pregnancy outcomes	In women, one example is premature deliveries. For this outcome, a primary driver is childhood sexual abuse.	[45]
Neural wiring alterations/Cognitive impairment	Neural rewiring appears to be associated with a cadre of neurobehavioral alterations.	[46,47]
Social/Emotional Impairment	This can range from mild social interaction issues to trauma-associated body disassociation.	[48,49]
Elevated risk of suicide	This often co-occurs with major depressive disorder.	[50,51]
HPA axis dysregulation	This recent review describes a neuroimmune regulation model for the resulting HPA hyperactivity.	[52,53]
Complex Post-Traumatic Stress Disorder (C-PTSD). This disorder is defined in the World Health Organization International Classification of Diseases 11th Revision [54]	The C-PTSD is a specialized category of PTSD that stems from childhood trauma.	[55,56,57]
Microbiome dysbiosis	Dysbiosis of the gut microbiomes from ACEs appears to lock in systems biology based dysfunctional responses to later life stress.	[58,59]

**Table 2:** Examples of Ealy Life Adverse Events, Inflammation, and Specific NCDs/Chronic Disorders (CDs).

Period of Early Life	Biomarkers (Population Subset)	NCD/CD Elevated Risk and/or Inflammation	Reference(s)
Early life adverse event	Fear as a marker of intensity	Irritable Bowel Syndrome	[63]
Adverse childhood event	Ace-induced immune programming appears to be a significant factor.	Pediatric Asthma	[12,64]
Adverse childhood experiences	Cross-sectional study demonstrating a strong positive association of developmental programming of adult asthma.	Adult-onset asthma	[65]
Early life bereavement	In Women	Transgenerational early onset asthma	[66]
Adverse childhood event	Insulin resistance; above average BMI.	Adult obesity	[67]
Adverse childhood event	Arterial stiffness	Cardiovascular Disease	[68]
Adverse childhood experiences	Female psoriasis patients had more ACEs than controls or male patients.	Psoriasis	[69]
Adverse childhood experiences	Evaluated in adulthood and specifically associated with other ACE comorbidities (e.g., depression).	Coronary heart disease	[70]
Traumatic experiences	Patients had a higher number of traumatic experiences than control groups.	Type 2 Diabetes	[71]
Childhood traumatic stress	Retrospective cohort study of 15,357 adults in San Diego, CA.	Elevated risk of hospitalization with adult autoimmune disease (among 21 diseases)	[72]
Child abuse	A study of 36,152 women from the Black Women’s Health Study.	Systemic Lupus Erythematosus	[73]
Child abuse	A longitudinal cohort study of 67,516 women from the Nurses’ Health Study II.	Systemic Lupus Erythematosus	[74]
Childhood trauma (and lifetime trauma)	A case-controlled study involving 71 HS patients and 213 controls.	Hidradenitis suppurativa (HS)	[75]
Early life adversity	Part of the British National child development study (NCDS), a prospective birth cohort study using 1958 births.	Cancer: elevated risk of early onset cancer (before age 50) among women	[76]
Adverse childhood experiences	Meta analysis for any cancer	Cancer	[16]
Adolescent sexual abuse	Analysis of immune parameters in patients (*n* = 33) with a history of childhood sexual abuse vs. controls (*n* = 10).	Dysregulation of the immune system (elevated eosinophils, reduced Th1 cytokines) among adolescents with PTSD	[77]
Childhood trauma	Results from the Netherlands Study of Depression and Anxiety (NESDA), *n* = 2778	Depression and Anxiety	[78]
Childhood trauma	A longitudinal cohort study of 1419 British children, Adolescent inflammation was a biomarker.	Psychotic experiences	[79]
Adverse childhood experiences	Data analysis from the China Health and Retirement Longitudinal Study.	Adult Depression	[80]
Childhood trauma	Review article concluding that childhood trauma increases the risk BP.	Bipolar Disorder (BP)	[81]
Adverse childhood experiences	Cross sectional analysis of 1223 participants aged 65 or older. ACEs increased the likelihood of a positive diagnosis for dementia in later life.	Dementia	[82]
Childhood maltreatment	Case controlled study of pairs.	Endometriosis	[83]
Childhood trauma	A cross-sectional study involving 279 nurses from six hospitals in South Korea.	Sleep disorders	[84]
Adverse childhood experiences	A cross-sectional study of 22,403 adults from the 2011 Behavioral Risk Factor Surveillance System.	Short sleep duration	[31]
Childhood trauma	A cross sectional study from among 182 patients referred to a geriatric mental health facility.	Multidimensional frailty	[85]
Childhood trauma	Analysis of 655 in-patients with severe PTSD.	Post-traumatic stress disorder (PTSD)	[86]
Childhood trauma or high ACE score	Study of epigenetic methylation patterns of specific genes following childhood trauma as a predictor of PTSD among combat troops; 170 participants; Of seven candidate genes, three showed a lower methylation pattern associated with PTSD development following childhood trauma and/or a high ACE score.	Post-traumatic stress disor-der (PTSD)	[87]
Childhood trauma	Study of 155 adults with PTSD following childhood trauma; imagery rescripting (ImRs) and eye movement desensitization and reprocessing (EMDR) were found to be useful treatments of this cohort.	Post-traumatic stress disor-der (PTSD)	[88]
Adverse childhood experiences	Data from 108 low-income African-American adolescents; Shortened telomere length, elevated C-reactive protein levels, and increased waist circumference were all biomarkers from path analysis.	Increased cardiometabolic risk	[89]
Childhood maltreatment	A cross-sectional study in of 561 individuals (ranging between 6 and 14 years of age) from a large prospective community school-based study, i.e., the Brazilian High-Risk Cohort (HRC); Shortened telomere length (males only).	Shortened telomere length in males	[90]
Early life adversity	Study of 93 preschool-age children; early life adversity associated with increased salivary inflammatory cytokine biomarkers.	Increased salivary inflammation (based on salivary cytokine profiles)	[91]
Adverse childhood experiences	Analysis of 8810 members of the 1958 British birth cohort; 12 ACE criteria were used and three inflammatory markers were assessed.	Elevated inflammation was found associated with ACEs. While even some low ACE scores within specific categories of events were associated with increased inflammation in mid-life, polyadversity led to the greatest inflammation increases. Specific combination of ACEs may be more important than others pertaining to elevated inflammation.	[92]
Parent-child separation	574 adolescents were evaluated. Persistent parent–child separation experiences led to significantly increased biomarkers of inflammation	Increased inflammatory burden	[93]
Multi-hit early life events	This is a rodent study using C3H/HeN mice of both sexes. Sex-specific effects in the adults were evident.	Microbiota alterations; Behavioral outcomes with difference in the adults based on sex.	[94]

**Table 3:** Some Recent Examples of ACE Outcomes and Pain.

Outcome(s)	Details	References
Pain Functional Interferences; Changes in Threat Appraisal	A study of *n* = 114. Males only showed significant differences.	[43]
Chronic pain	This was a cross-sectional analysis of the 2016–2017 National Survey of Children’s Health. There were 48,567 child participants ages 6 to 17. A significantly higher prevalence of children with one or more ACEs experienced chronic pain vs. children with no ACEs.	[96]
Persistent pain	This is a proposed study protocol for a systematic review and meta-analysis of ACEs and persistent pain in adults.	[40]
Adolescent pain	A study of 219 adolescents in rural China; Separation; host genetic variations considered; a higher pain score was found among adolescents separated from both parents.	[41]
Chronic Pain	A survey study of 8140 employees of City of Helsinki, Finland ages 40–60 years old. It included seven categories of ACEs. This was part of the Helsinki Health Study.	[42]
Cortisol levels are a useful biomarker	Review article on the social ecology of early life adversity.	[44]

**Table 4:** Recent Examples of ACE and Substance Misuse/Abuse/Addiction.

Outcome	Details	Reference
Substance addiction	A qualitative study from Iceland in males who had experienced child abuse.	[110]
Substance misuse	Meta-analysis; A study of misuse in young people found that ACE can drive male misuse of tobacco.	[111]
Substance abuse	A study using data from the 2016–2019 National Survey of Children’s Health; During childhood; Seven ACEs were included (but not child maltreatment).	[46]
Problematic alcohol and/or tobacco use	A longitudinal study of 1179 youths of Lain American origin or descent. The authors concluded that the results suggest a predictive relationship exists for ACEs and specific abused substances.	[112]
Binge drinking	Data were obtained for 80,391 individuals from the Behavioral Risk Factor Surveillance System (2011–2017). Effects of combined ACEs were noted as were differential impact by sex.	[113]
Polysubstance use	A longitudinal study with *n* = 2880; Latent transition analysis was used to compare. Youths in the ACE+ group were more likely to have more categories of substance abuse and to not transit out of that as readily as ACE–youths.	[37]
Prescription drug misuse	A survey-based study among California college students found significant increased misuse for all prescriptions with increased odds for each added ACE. Stimulant misuse among identifying Asian/Pacific Islander (API) and Hispanic students with ACEs was noted.	[114]

**Table 5:** Examples of Circadian Disruption and Adverse Outcomes.

Chronic Disorders	Reference(s)
Alcohol addiction	[123]
Mood disorders	[124]
Depression	[125]
Pain	[126]
Chronic inflammation	[127]
Neurodegeneration	[127]
Metabolic dysregulation and disease	[128,129]
Sleep quality/disorders	[130]
HPA axis dysregulation	[131]
Elevated risk of atherosclerosis	[132]
Diseases of the skin	[133]
Tumorigenesis	[134]
Increased risk of asthma	[135]
Promotion of allergic diseases	[136]

**Table 6:** Microbiota and Circadian Medicine.

Circadian-Related Condition	Effects	Reference
Gut microbiota comparisons among circadian-associated sleep disruption	In this cross-sectional study, short sleepers (less than six hours) were found to have significantly lower *Sutterella* and significantly elevated *Pseudomonas* when compared with the gut microbiota of long sleepers.	[137]
Immune and metabolic homeostasis	A review of how cyclic metabolism of gut microbiota of short-chain fatty acids, tryptophan metabolites, and bile acids significantly affect the status of the microimmunosome (barrier function, immune cell balance, control of tolerance and inflammation).	[138]
Microbial oscillators of dietary cues, circadian clock, and metabolism	This review article describes how the microbiome controls chronometabolism and host metabolic phenotypes via microbial metabolites (e.g., short-chain fatty acids), microbial components (e.g., flagellin), and nuclear receptors.	[139]
Microbes and circadian medicine	This review focuses on (1) inherent rhythms among microbiota, (2) the cross-talk between the circadian clock and microbiota and (3) how the combined interactions either produce homeostasis or dysbiosis, immune and physiological dysfunctions and pathology.	[140]
Disrupted circadian rhythms	Intermittent fasting aligns circadian rhythms via the gut microbiome.	[141]
Circadian misalignment	Proof of concept that disrupted circacadian rhythm affects the oral microbiome composition and metabolic function as well as functional pathways affecting immunity.	[142]

**Table 7:** Examples of Microbiota Status and Sleep in Humans.

Details	Reference(s)
A *Lactobacillus plantarum* probiotic was found to aid deep sleep.	[144]
*Bifidobacterium longum* supplementation provided improved sleep during heightened stress.	[145]
A probiotic mix improved sleep quality.	[146]
Gut microbiome status including altered metabolism affects sleep.	[147]
Probiotic supplementation improved sleep among postoperative cancer patients.	[148]
Sleep quality improved with a probiotic complex.	[149]
Specific gut microbiota predicts short vs. normal sleep.	[150]
Gut microbiome dysbiosis can produce an overactive bladder which disrupts sleep.	[151]
A review of gut microbiome status including altered metabolism and the impact on sleep.	[152]
A review of sleep disorders and gut dysbiosis and how they go together.	[153]
A review of sleep disruption and microbiome metabolic dysregulation.	[154]
A review of using microbiota to control the sleep–wake cycle as we age.	[155]

**Table 8:** Inflammation, Oxidative Damage and Telomere Shortening.

Inflammatory Damage and Telomere Status	Reference(s)
Linking telomere length, inflammation, and gut dysbiosis.	[158]
Oxidative stress damages telomeres and mitochondria.	[159]
Early life factors program both inflammation and telomere shortening.	[160]
Chronic inflammation generates immune aging and cross-talk with the telomere complex.	[161]
Link between telomere shortening and tissue inflammation.	[162]
Proposal that inflammation, telomere length, and microbiota may form a loop.	[163]

**Table 9:** Examples of Microbiome-Altering, Inflammation Reducing, and/or Anti-Aging Supplements.

Supplement	Study/Effect	Reference(s)
Hyaluronic acid	Provides extracellular matrix support, acts as a form of prebiotic for gut microbiota, restores gut barrier function within the microimmunosome, acts as a therapeutic/prebiotic to rebalance skin microbiota, acts as an anti-inflammatory agent; alters macrophage polarization within the microimmunosome.	[167,168,169,170,171,172]
Red Ginseng	Reported dual regulation of oxidative stress and increases in *Bifidobacteria* and *Akkermansia* gut bacteria.	[173]
*Limosilactobacillus fermentum* strains	Supplementation with the probiotic mix reduced both inflammation and oxidative stress.	[174]
Polyphenols from Fu brick tea	Increases in core gut bacteria *Akkermansia muciniphila*, *Alloprevotella*, *Bacteroides*, and *Faecalibaculum;* improved barrier function, reduced oxidative stress in the intestine.	[175]
*Lactobacillus salivarius* FDB89; *Bacillus licheniformis* *Lactobacillus gasseri* SBT2055, *Lactobacillus gasseri* SBT2055, *Lactococcus lactis* subsp. *cremoris* H61, *Lactococcus lactis* subsp. *lactis* JCM 5805, *Lactococcus lactis* subsp. *lactis* strain Plasma, *Lactobacillus plantarum* HY7714	A review article in 2018 listing studies with seven distinct probiotics that that were found to have anti-aging properties when administered. The first six were in model systems and the last one (*Lactobacillus plantarum* HY7714) was in human volunteers directed toward skin.	[176]
*Lactobacillus fermentum* DR9 *Lactobacillus paracasei* OFS 0291 *L. helveticus* OFS 1515	Evaluation of three probiotics strains (*Lactobacillus fermentum* DR9, *Lactobacillus paracasei* OFS 0291 and *L. helveticus* OFS 1515 in rats for anti-aging effects in bone. Of the three, *Lactobacillus fermentum* DR9 was the most effective.	[177]
*Lb. rhamnosus* CRL981, *Lb. plantarum* CECT 748T, *Lactobacillus* sp. Niu-O16, *Lb. rhamnosus* INIA P540 *Ent. faecalis* INIA P333, *Lb. mucosae* EPI2, *Ent. faecium* EPI1, *Finegoldia magna* EPI3, and *Veillonella* sp. EP, *Lactococcus garvieae* 20–92, *B. breve* 15700 and *B. longum* BB536, *B. adolescentis* INIA P784, *Gordonibacter urolithinfaciens* and *Gordonibacter pamelaeae* DSM 19378T	A review of 12 different probiotic strains or mixtures that improve the senescent immune system via phytoestrogen metabolism.	[178]
A variety of probiotics and synbiotics among 16 studies included in this meta-analysis	A review article and meta-analysis of 16 studies of probiotics or synbiotics on diabetic patients. The results suggested that these supplements can improve biomarkers of inflammation and/or oxidative stress.	[179]
*Lactobacillus plantarum* GKM3	A study on mice found that this probiotic promotes longevity, reduces oxidative stress in the brain, and supports memory retention.	[180]
*Streptococcus thermophilus* TCI633	A study on humans showing the anti-aging effects of this orally administered probiotic on skin.	[181]
Human trial with a symbiotic preparation *(Lactobacillus paracasei*, *Bifidobacterium longum*, *Bifidobacterium breve*, inulin, and fructooligosaccharide)	A randomized human trial of 12 weeks duration on Thai obese adults. Among the changes seen, both inflammatory and oxidative stress biomarkers improved with the symbiotic supplement.	[182]
*Bifidobacterium longum* and the prebiotic, galacto-oligosaccharide	A study on mice found that this orally administered symbiotic combination protected against UVB-photoaging of skin.	[183]
*Weissella confusa CGMCC 19*,*30*	A study on the bacterial infection C. elegans model found that this orally-administered probiotic increased lifespan, improved immunity, and reduced oxidative stress.	[184]
*Lactobacillus paracasei* GKS6 and *Bifidobacterium lactis* GKK2 were examined independently.	A fourteen-week study on aged mice used a two-bacteria combination probiotic to examine anti-aging effects. The results showed both probiotics significantly increased antioxidant activity thereby reducing oxidative stress. *B. lactis* had a positive effect on muscle building.	[185]
*Lactobacillus plantarum* HY7714	This is a detailed mechanistic study on cell lines investigating the molecular mechanisms through which this probiotic bacterium protects skin from aging. Among the changes were reduced inflammation and oxidative stress and improved tight junction status.	[186]
*Lactobacillus rhamnosus* KCTC 5033 (a paraprobiotic group was also included in this study)	Improved skin hydration on the necks of middle-aged women following a 12-week duration trial vs. controls.	[187]
*Lactobacillus plantarum* HY7714	A randomized, double blind, placebo-controlled study of 12 weeks duration was conducted among 100 middle-aged volunteers with dry skin. The probiotic supplementation improved skin elasticity and hydration and reduced wrinkle depth.	[188]
*B. longum* BB68, *L. gasseri* SBT2055, *L. fermentum* MBC2, *B.* *infantis* ATCC15697, *B. subtilis* PXN21, *L. brevis* OW38, *L. paracasei* PS23, *L. paracasei* K71, *L. plantarum* AR501, *L. helveticus* KLDS1.8701, *L***.** *plantarum* C29, *L. plantarum* NDC 75017, *L. fermentum* DR9, *B. breve* B-3, *L. casei* Shirota	A review article including the results from 16 different probiotic strains that produce the anti-aging outcomes. The review article also proposes a new term “gerobiotics” for supplements specifically designed to produce anti-aging effects.	[189]
*Lactococcus lactis* subsp. *cremoris* C60	Probiotic supplementation of IL-18 deficient mice restored a dendritic cell promoted T-cell-based immune function whose decline is connected to immune senescence.	[190]
*Akkermansia*	This survey study of the American Gut Project database confirms that *Akkermansia* is a major target for anti-aging protection.	[191]
A mixture of a specialized *Lactobacillus kefiri* strain product and a minor component yeast strain	A Kefir-derived product was found to reduce oxidative stress in mice.	[192]
*L. salivarius* AP-32	Probiotic supplementation in rats increased antioxidant capacity and was neuroprotective.	[193]
*Lactobacillus plantarum* DR7, *Lactobacillus fermentum* DR9, *Lactobacillus reuteri* 8513d	*Lactobaccillus* probiotic strains protected against telomere shortening in a rat aging model.	[194]

## Data Availability

Data discussed in this review article are available via the cited references.
